# Mass and Charge Transport in Li_1−δ_CoO_2_ Thin Films—A Complete Set of Properties and Its Defect Chemical Interpretation

**DOI:** 10.1021/acs.chemmater.2c02614

**Published:** 2022-11-21

**Authors:** Andreas E. Bumberger, Claudia Steinbach, Joseph Ring, Juergen Fleig

**Affiliations:** Institute of Chemical Technologies and Analytics, TU Wien, Getreidemarkt 9/164-EC, Vienna1060, Austria

## Abstract

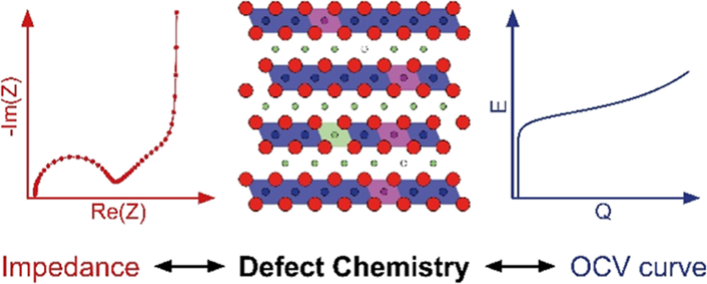

Lithium insertion materials are an essential class of mixed ionic and electronic conductors, and their electrochemical properties depend on the resistive and capacitive interplay of ions and electrons. However, complete sets of the corresponding elementary material parameters, that is, composition-dependent ionic and electronic conductivity, chemical capacitance, and charge-transfer resistance, are rarely reported for lithium-ion battery electrode materials. Moreover, the interpretation of these properties from a defect chemical point of view is not very common. In this work, the impedance of sputtered Li_1−δ_CoO_2_ thin films is analyzed to extract the fundamental electrochemical properties as a function of state-of-charge (SOC). Within the accessible SOC range, the charge transfer resistance and ionic conductivity vary by more than 1 order of magnitude. The chemical capacitance determined from impedance spectra agrees excellently with the differential capacitance from charge/discharge curves, and, in the dilute regime, even matches the absolute values predicted by defect thermodynamics. The evolution of lithium diffusivity along the charge curve is deconvoluted into the separate contributions of ionic conductivity and chemical capacitance. Finally, we apply the principles of defect chemistry to evaluate the observed trends in terms of lithium activity and point defect concentrations and provide a tentative defect model that is consistent with our results. The consistency of impedance measurements, cycling data, and thermodynamic theory highlights the key role of the chemical capacitance as a powerful material descriptor and emphasizes the relevance of defect chemical concepts for all lithium insertion electrode materials.

## Introduction

Cathode materials for rechargeable Li-ion cells continue to be the focus of many research efforts in both academia and industry. With the high demands placed on modern Li-ion batteries with respect to their charging speed and discharge power density, the investigation of Li transport kinetics through the various cell components plays a vital role in optimizing cell performance. However, owing to the morphological and compositional complexities found in porous bulk electrodes, the investigation of their kinetic properties is far from trivial. Measurement approaches range from time-dependent voltage or current measurements, such as the galvanostatic intermittent titration technique,^1−5^ to sophisticated transmission line models, taking account of both electrolyte and electrode.^6^ However, as pointed out recently by Chueh,^7,8^ the transport properties, such as Li diffusion coefficients, deduced from different studies often differ by orders of magnitude.

One of the main reasons for this variance is the fact that several time constants are involved in the time- or frequency-dependent responses of highly porous intercalation electrodes. Moreover, even if extracted from geometrically simple samples such as thin films, the chemical diffusion coefficient of Li (*D̃*) is not the only relevant parameter for the charge and discharge properties of electrode particles. Rather, the kinetics of an individual electrode particle is defined by (i) the interfacial Li exchange reaction with the electrolyte, (ii) the ambipolar conductivity within the mixed conducting electrode material itself, which comprises the ionic and electronic conductivities σ_ion_ and σ_eon_, and (iii) the chemical capacitance *C*_chem_, as discussed in more detail below. The chemical diffusivity itself is, however, not an elementary property of the electrode material but a composite parameter of ambipolar conductivity and chemical capacitance.^9−11^ To further complicate things, a battery electrode traverses a broad continuum of operating points or thermodynamic states during every charge/discharge cycle, each being defined by its characteristic Li chemical potential and electrochemical transport parameters. The elucidation of these electrochemical parameters as a function of state-of-charge (SOC) is therefore a notoriously difficult task and has mostly been limited to the evaluation of the chemical diffusion coefficient *D̃* from the inverse time constant of the observed diffusion processes, without separate consideration of its resistive and capacitive components.^12−18^

It is also frequently overlooked in this context that *C*_chem_, which describes the differential variation of Li stoichiometry with the chemical potential, is not only related to the slope of the coulometric titration curve and the energy density but also directly impacts solid-state ambipolar diffusion and hence the power density.^10^ Consequently, *C*_chem_ can be extracted from AC impedance spectra as well as from DC titration curves, and these values should coincide. However, this connection is rarely made, which is mirrored by the context-dependent labels that are used throughout the battery literature to denote the variation of charge with potential. In order to describe the impedance of mixed conducting electrodes, a one-dimensional transmission-line equivalent circuit was suggested by Jamnik and Maier,^19−23^ giving access not only to *C*_chem_ but also to the transport properties σ_ion_, σ_eon_, and the interfacial charge-transfer resistance *R*_ct_. Applying this model (or simplified versions) to a one-dimensional electrode system, such as a thin-film electrode, should thus enable the analysis of all elementary material parameters and help in understanding the stoichiometry-dependent Li intercalation process into an electrode material. Interestingly, such a transmission-line-based analysis of impedance spectra of mixed conducting thin-film electrodes is quite common in the field of high-temperature solid oxide cells^23−26^ but virtually unknown for thin-film electrodes in Li-ion batteries.

In this contribution, we present a comprehensive impedance study of polycrystalline Li_1−δ_CoO_2_ (LCO) thin films on Pt for a wide stoichiometry range (0 ≤ δ ≤ 0.4) that also includes the rarely investigated low-potential region up to 3.9 V versus Li^+^/Li, where the most pronounced changes of the electrochemical properties are observed. Based on these measurements, we discuss three important aspects of Li intercalation into Li_1−δ_CoO_2_. First, we analyze the variation of σ_ion_, *C*_chem_, *D̃*, and *R*_ct_ with the Li chemical potential and discuss their relative contributions to the overall electrode kinetics. Second, the role of *C*_chem_ in AC and DC measurements is discussed and experimentally validated, and its overarching significance for ambipolar transport is considered. Third, we provide a defect chemical perspective on the observed trends of σ_ion_, *C*_chem_, *D̃*, and *R*_ct_ in terms of Li activity. Special emphasis is thereby put on the rarely investigated SOC region close to full Li stoichiometry,^16^ where dilute solution thermodynamics can be applied to describe the electrochemical properties in terms of their dependence on point defect concentrations. All these concepts are herein specified for LCO but are applicable to all battery electrode materials that are based on ion insertion.

## Basic Considerations on *C*_chem_ and Impedance Models

### Chemical Capacitance

There appear to be three separate contexts within which the chemical capacitance^10^ is used for describing Li-ion battery electrodes, each using different labels that reflect the purpose at hand. The first and, in studies on porous bulk electrodes, the most widespread use is the monitoring of gradual material changes and phase transitions as a function of cell voltage and cycle number.^27−37^ In this context, the chemical capacitance is termed *differential capacity*, *incremental capacity*, or d*Q*/d*V* and is almost exclusively obtained via the differentiation of galvanostatic charge curves. Its appearance in impedance spectra is usually not considered, presumably because capacitances in such electrodes are too large to exhibit blocking (finite-space) behavior within reasonable measuring frequencies. This is also reflected in the transmission-line equivalent circuits used to describe porous electrodes. In most cases, they contain no open Warburg element or capacitance in series to the charge-transfer resistance and hence implicitly assume an infinitely large chemical capacitance.^38−41^

The second way chemical capacitances are used in the literature, which is predominant in impedance studies on thin-film electrodes,^12,14,15,42^ is as an input parameter for the extraction of diffusion coefficients from the semi-infinite (45°) diffusion regime. These studies still mostly refer to the *incremental capacity*, although they label it d*x*/d*E* (or similar) in calculations and recognize it as a parameter that is needed for the calculation of diffusion coefficients from impedance data.^43^ However, these reports still treat the chemical capacitance mainly as an empirically derived property of the charge curve rather than an elementary material parameter that relates back to thermodynamics and defect chemistry. This is evidenced by the continued use of the term *incremental capacity* and the approach of differentiating charge curves adopted from bulk studies. Interestingly, also in the field of Li insertion thin films, chemical capacitances are barely ever extracted from impedance spectra,^44,45^ which is surprising, given that films are typically thin enough to show a finite-space (90°) behavior within the mHz range. Theoretical works on the finite-space diffusion impedance of thin films already refer to a (diffusion- or low-frequency-limiting) *capacitance* rather than a *capacity*,^43,46,47^ explicitly recognizing its role as a capacitive element in impedance measurements. However, this perspective does not seem to be adopted by the previously cited studies that apply those theories to battery electrodes.

Finally, the third established context of chemical capacitance is based on an atomistic analysis of the basic relation^19,48^
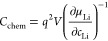
1which describes the electrochemical Li storage in an electrode with bulk volume *V* (e.g., a dense thin film with thickness *L*, deposited on area *A*) as a function of the equilibrium Li concentration *c*_Li_, with the elementary charge *q*. This approach to *C*_chem_ is particularly helpful if dilute defect chemical considerations are still valid. The Li chemical potential μ_Li_ is related to the Li activity *a*_Li_ via
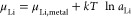
2with *k* and *T* denoting Boltzmann’s constant and temperature, respectively, and μ_Li,metal_ being the chemical potential of metallic Li. For a given chemical potential difference between Li metal (*a*_Li,metal_ = 1) and cathode, the cell voltage *E* can be defined as
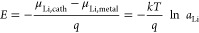
3For a more detailed analysis of μ_Li_, its separation into electronic and ionic contributions is highly useful. Analogously to the defect chemical considerations of mixed conducting oxides in solid oxide fuel cells, Brouwer diagrams thus come into play, where point defect concentrations and their dependence on Li activity are derived from laws of mass action and chemical potentials. However, as emphasized by Maier,^49−51^ such concepts have hardly permeated the field of Li electrode materials, despite being very helpful for a more detailed understanding. For this reason, the concept of chemical capacitance and its universal presence in charge/discharge curves, impedance spectra, and ambipolar mass and charge transport has rarely been addressed in Li-ion literature. It is also noted that the interfacial pseudocapacitances of transition-metal oxides include local compositional and oxidation state changes and are thus related to the above definition of chemical capacitances. A detailed discussion of pseudocapacitances can be found, for example, in ref (52).

In this study, we will investigate the electrochemical behavior of Li_1−δ_CoO_2_ in the stoichiometry range 0 ≤ δ ≤ 0.4, where Li is reversibly deintercalated via oxidation of the material, starting at initially full stoichiometry (δ ≈ 0). We may consider Li vacancies V_Li_^′^ and electron holes h^•^ as the relevant defect species, at least as long as defect chemical concepts are applicable to intercalation processes.^51,53^ In order to describe the deintercalation in terms of (initially) dilute point defects, it is convenient to reconsider the chemical potential of atomic Li in Li_1−δ_CoO_2_ (eq 2) in terms of charged species, that is, as μ_Li_ = μ_Li^+^_ + μ_e^–^_, which, for defects, transforms into^49^
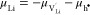
4with
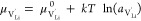
5
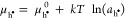
6The defect activities *a*_*i*_ are related to the corresponding defect concentrations *c*_*i*_ by the activity coefficients γ_*i*_, for example, for Li vacancies by
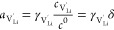
7where μ_*i*_^0^ denotes the standard chemical potential of the respective species, and defect concentrations are referred to the concentration of formula units *c*^0^. Thus, the nonstoichiometry δ corresponds to . Under the assumption of dilute conditions and absence of charge trapping, the defect activity coefficients γ_*i*_ → 1 and eq 1 can be evaluated as^54^
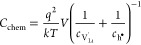
8This reflects the behavior of *C*_chem_ as a serial double capacitor with an effective capacitance that is determined by the concentrations of both ionic and electronic charge carriers. The value of *C*_chem_ is primarily determined by the minority carrier concentration. In general, the interplay of several defect chemical effects determines the minority species, including intrinsic ionic and electronic disorders, doping, or site changes. Assuming , for example, the chemical capacitance becomes
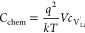
9

### Transmission Line Model, Conductivity, and Chemical Diffusion

**Figure 1:**
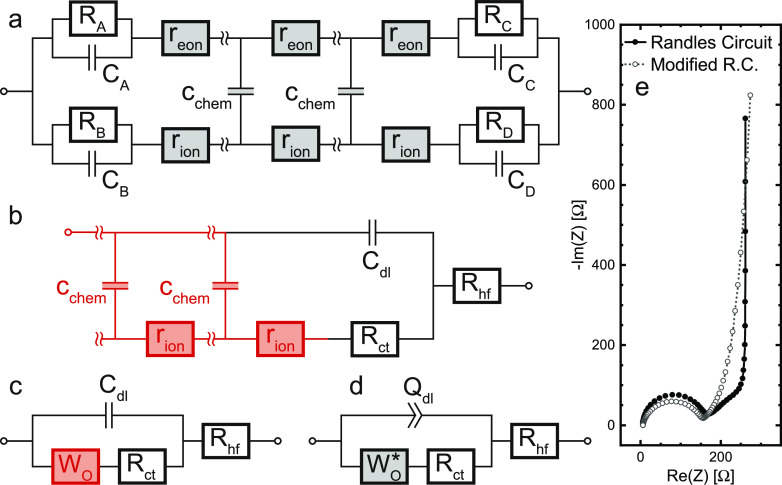
(a) General one-dimensional transmission line of a mixed conductor consisting of electronic/ionic resistive elements and chemical capacitors, including terminal *R*/*C* elements. (b) Simplified transmission line for a one-dimensional Li storage electrode extended by a serial high-frequency offset resistance. The obtained circuit is fully equivalent to (c) Randles’ circuit. (d) Modified Randles’ circuit with an anomalous diffusion element and nonideal double-layer capacitance. (e) Simulated impedance response of circuits (c,d) for *R*_hf_ = 5.66 Ω, *R*_ct_ = 143.6 Ω, *C*_dl_ = 0.12 mF, *R*_ion_ = 327 Ω, and *C*_chem_ = 209 mF. For the modified Randles' circuit, the interfacial capacitance was modeled as a constant-phase element, with *Q*_dl_ = 0.12 mF s^–0.15^ and the corresponding constant-phase exponent of 0.85, and the open Warburg element was replaced by an anomalous finite-space diffusion element *W*_o_^*^ with a nonideality factor of α = 0.72 (see eq 19).

In a one-dimensional situation, the electrochemical bulk properties of a mixed ionic electronic conductor at a fixed stoichiometry can be described by a transmission line (Figure 1a) consisting of two resistive rails (ions and electrons) and capacitive connections representing the local chemical charge storage, with *R*_*i*_ = ∑*r*_*i*_ and *C*_chem_ = ∑*c*_chem_.^19−23^ The corresponding bulk properties are thus fully characterized by the three elementary parameters σ_eon_, σ_ion_, and *C*_chem_. By definition, the ionic or electronic conductivity of a material is given as
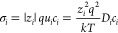
10with the charge number *z*_*i*_, carrier mobility *u*_*i*_, carrier diffusion coefficient *D*_*i*_, and equilibrium concentration *c*_*i*_ of charge carrier *i*. For mixed conduction, the ambipolar conductivity σ̃ can be defined as
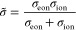
11and is related to the ambipolar (chemical) diffusion coefficient via^10^
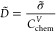
12where *C*_chem_^*V*^ is the volume-specific chemical capacitance, with *V* = *AL*, *A* = area, and *L* = thickness. In the case of high electronic conductivity (σ_eon_ ≫ σ_ion_), we have σ̃ ≈ σ_ion_, and thus
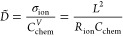
13where *R*_ion_ is the total ionic resistance of the thin film in the direction of transport. The ionic conductivity may also be expressed in terms of defects (V_Li_^′^), that is,
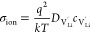
14and from eqs 9, 10, and 13, we then get  for .

### Impedance of a Li Intercalation Electrode

The impedance response of a thin film battery electrode is commonly described by Randles’ circuit, which is shown in Figure 1c.^55,56^ This intuitively constructed equivalent circuit can also be derived directly from the general transmission line introduced above (Figure 1a) by applying the appropriate boundary conditions and simplifying assumptions.^20^ First, we assume that σ_eon_ ≫ σ_ion_, and therefore the electrical potential gradients within the material can be neglected. This means that electronic resistances *r*_eon_ in the transmission line can be neglected and the corresponding rail replaced by a short circuit. At the bottom of the thin film, the current collector presents an ion-blocking boundary (*R*_B_ → ∞, *C*_B_ → 0) that is reversibly transmissive for electrons (*R*_A_ → 0, *C*_A_ → ∞). The liquid electrolyte in contact with the thin-film surface is electron-blocking with an interfacial double-layer capacitance (*R*_C_ → ∞, *C*_C_ → *C*_dl_) but allows the reversible transport of Li ions across the corresponding charge-transfer resistance (*R*_D_ → *R*_ct_). As the interfacial capacitance is assumed to be located on the electronic rail terminal, the remaining capacitance at the ionic terminal is neglected (*C*_D_ → 0). Upon adding a serial high-frequency resistance *R*_hf_ to account for an Ohmic offset due to the electrolyte and other cell components, the resulting circuit (Figure 1b) is identical to Randles' circuit, with the open Warburg element *W*_o_ being equivalent to the reflective transmission line marked in red. The corresponding expression *Z*_*W*_o__(ω) is given by
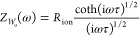
15and describes the impedance of one-dimensional finite-space diffusion, as derived from Fick’s laws.^43^

As can be seen from eq 15, the impedance of the Fickian diffusion process is composed of a resistance *R*_ion_ and a time constant τ. The chemical diffusion coefficient *D̃* is the inverse time constant normalized by a geometrical factor
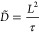
16The time constant itself is the product of the characteristic resistance and capacitance of the transport process, and in accordance with eq 13, it reads
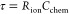
17

## Experimental Section

### Preparation of LiCoO_2_ Thin Films

One-side-polished sapphire (0001) single-crystal substrates with dimensions 10 mm × 10 mm × 0.5 mm (CrysTec, Germany) were sonicated in ethanol (absolute, VWR, Germany) prior to use. A bilayer of Ti/Pt (5/100 nm) was deposited on both sides of the substrate as a current collector via DC-sputtering at room temperature, a current density of 5 mA/cm^2^, and Ar pressures of 0.7 and 2.0 Pa, respectively. LiCoO_2_ thin films were deposited onto the polished side of the substrates via RF magnetron sputtering in a custom-built deposition chamber (Huber Scientific, Austria) at room temperature, a total pressure of 2.5 Pa in an Ar/O_2_ mixture (25% O_2_), power of 60 W, and a substrate-to-target distance of 8.5 cm. The LiCoO_2_ sputter target (diameter 2″) was obtained from Loyaltargets (China) and abrased with a fine sandpaper before each deposition to eliminate deviations from bulk stoichiometry at the target surface. The thickness of the LiCoO_2_ films was approximately 100 nm, as determined by a TEM thickness calibration, and the corresponding deposition rate was 0.5 nm/min. The as-deposited films were annealed in a muffle furnace in air at 700 °C for 5 h at a ramp rate of 10 °C/min. The samples were characterized by grazing-incidence X-ray diffraction (GID, ω = 3°), atomic force microscopy (AFM), and elemental analysis via inductively coupled plasma mass spectrometry (ICP-MS).

### Electrochemical Characterization

After annealing, the thin-film samples were transferred into an argon-filled glovebox (O_2_ and H_2_O levels < 0.1 ppm) and assembled into three-electrode test cells (PAT-Cell by EL-Cell, Germany) using a glass fiber separator (260 μm, EL-Cell), 80 μL of a standard organic electrolyte (1 M LiPF_6_ in a 1:1 mixture of ethylene carbonate and dimethyl carbonate, Aldrich), and a Li metal anode (0.6 mm, Goodfellow, Germany). All electrochemical measurements were carried out at room temperature on a BioLogic SP200 potentiostat with a built-in impedance analyzer. Directly after assembly, a cyclic voltammogram of the working electrode was recorded at a scan rate of 0.1 mV/s in the potential range of 3.7–4.0 V versus Li^+^/Li, followed by galvanostatic charge and discharge within the same potential range at a current of 7.16 μA (1 C). Subsequently, a series of potentiostatic impedance spectra (1 MHz to 1 mHz, 6 points per decade, and 10 mV amplitude) was recorded in 10 mV steps, ranging from 3.85 to 4.00 V (charge scan) and back to 3.85 V (discharge scan) versus Li^+^/Li. Before each measurement, a 15 min constant voltage step was applied to allow the thin film to fully equilibrate at the given potential. To distinguish irreversible degradation from the potential-dependent changes of material parameters, the hysteresis between charge and discharge scans was evaluated. As the hysteresis was substantial for the first set of measurements due to the initial changes of the thin film, the impedance series was repeated, including CV scans before and after, yielding much more stable trends. The spectra obtained after stabilization were then analyzed in detail.

## Results and Discussion

### LiCoO_2_ Thin Films

**Figure 2:**
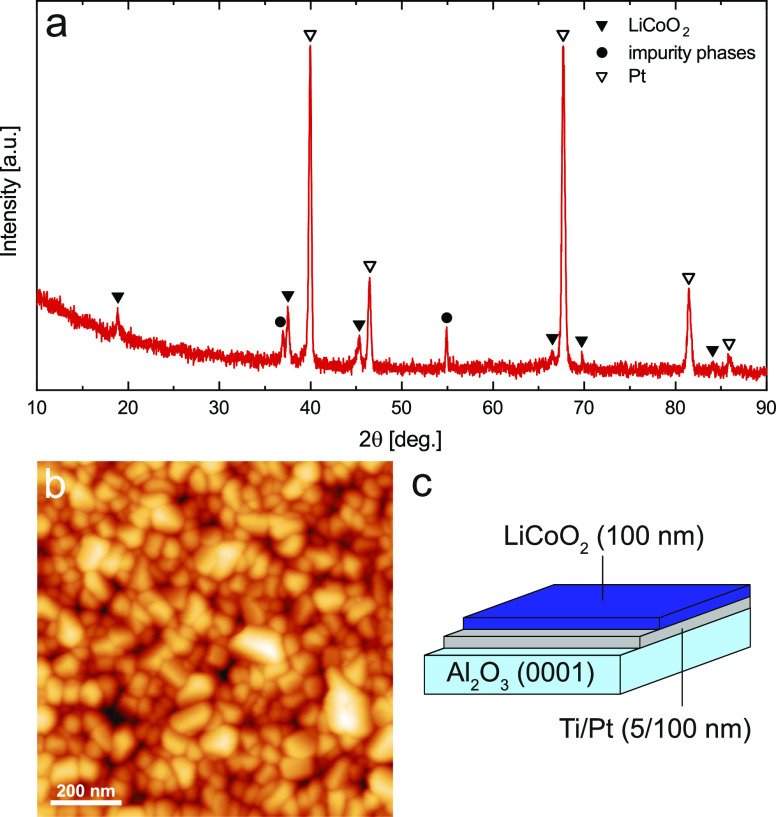
(a) GID pattern of LiCoO_2_ thin film at 3° incident angle. (b) AFM image revealing a rather homogeneous but polycrystalline film morphology with an RMS roughness of approximately 10 nm. (c) Schematic illustration of a LiCoO_2_ thin film deposited on a Ti/Pt-coated Al_2_O_3_(0001) single-crystal substrate.

The GID pattern of a typical LiCoO_2_ thin-film sample and its corresponding AFM image are displayed in Figure 2. The diffraction pattern shows the most characteristic LiCoO_2_ reflexes around 18.9° (003), 37.5° (101), and 45.3° (104). In addition, signals stemming from the Pt current collector can be clearly identified, as well as some impurity signals, most likely due to small amounts of Co_3_O_4_ in the sample. The AFM image reveals a reasonably homogeneous and polycrystalline morphology of the thin film, with an RMS roughness of approximately 10 nm. ICP-MS analysis of a thin film dissolved in concentrated hydrochloric acid yielded a Li/Co ratio of 0.95, indicating that the deposition of LiCoO_2_ was almost stoichiometric.

### DC Cycling

**Figure 3:**
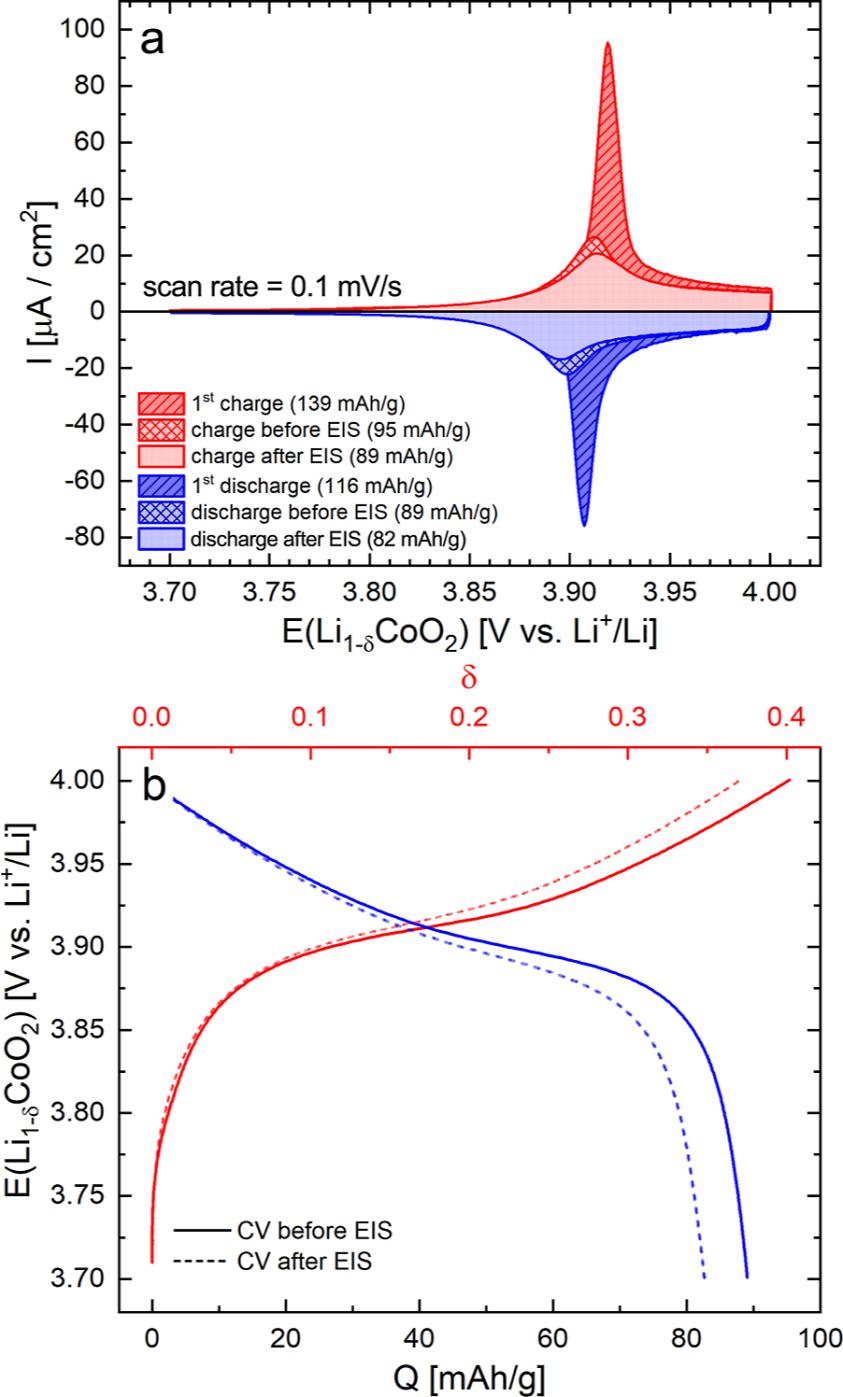
(a) CV curves of the LiCoO_2_ thin film in its pristine state (first charge/discharge) and in its stabilized state before and after EIS measurements. (b) Coulometric titration curves derived from stabilized CV curves. Nominal values of δ are obtained by assuming δ = 0.4 at 4.0 V for the CV scan before EIS. Charge values are normalized to the pristine thin-film mass.

Figure 3a shows the initial CV curve of the pristine sample (first charge/discharge) and CV curves after some initial changes and stabilization. More specifically, the latter correspond to the stabilized curves measured before and after the relevant series of impedance measurements discussed in this study. The film shows good electrochemical reversibility, with the initial discharge capacity of 116 mAh/g being in good agreement with the common literature values at this cutoff voltage.^13,18,57,58^ To evaluate the thin-film potential as a function of charge and Li stoichiometry, the CV curves are converted into the coulometric titration curves shown in Figure 3b. As is characteristic for LCO, the curves exhibit a plateau around 3.9 V, followed by a moderate and roughly constant slope up to 4.0 V. Nominal nonstoichiometry values δ are also shown in Figure 3b, which are deduced by assuming δ = 0.4 at 4.0 V^59^ rather than by relating the charge to the theoretical capacity of the film. This is also in accordance with the fact that the plateau region indeed corresponds to that of porous bulk electrodes, with the phase transition between two structurally very similar hexagonal phases in the range of 0.05 ≤ δ ≤ 0.25, followed by a single-phase (i.e. sloped) region starting at δ ≥ 0.25.^60−64^

Some degradation of the film capacity occurs between the initial cycle and the start of the impedance measurements, with a significant broadening of the CV current peak and the charge curve plateau becoming increasingly inclined. In terms of discharge capacity, the electrode drops to 89 mA h/g or 77% of its initial capacity. A similar broadening of the CV current (or differential capacity) peak accompanying this capacity loss has also been reported for porous bulk electrodes,^65^ and the frequently observed irreversible capacity losses of layered oxide cathodes are attributed to, among other factors, oxygen release and structural degradation toward denser spinel and rock salt phases, especially in the near-surface region, due to the thermodynamic instability of partially delithiated LCO.^66−70^ More importantly, however, there is only minor further degradation throughout the impedance measurements, meaning that the electrochemical properties extracted from the collected impedance spectra can be assumed to vary reversibly with the electrode potential.

**Figure 4:**
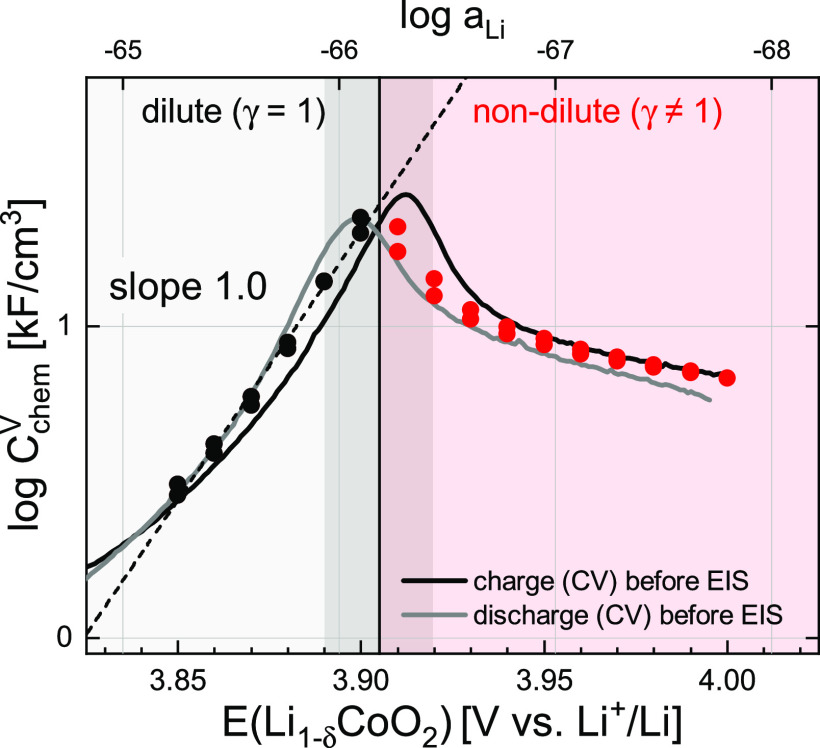
Chemical capacitance from CV scans (continuous lines) and impedance fits (discrete points) as a function of electrode potential and Li activity according to eq 2. The dilute to nondilute transition region 3.89–3.92 V is marked in dark around a central line at 3.905 V. The peak value of *C*_chem_ at this transition potential is around 29 kF/cm^3^. A linear fit for the dilute region is shown as a dotted line.

The DC data can now be used to determine the chemical capacitance (differential capacity) as a function of electrode potential. In the context of porous bulk electrodes, differential capacity curves are typically obtained via differentiation of galvanostatic charge curves from long cycling experiments, which require galvanostatic conditions to simulate a constant load. However, we perceive CV curves as a more suitable starting point when galvanostatic conditions are not required, as is the case for thin-film studies. This is because the differentiation of galvanostatic data often yields discontinuous and noisy capacitance curves due to the slight potential fluctuations in the original data. As a result, these data require smoothening prior to their conversion into differential capacity values.^27^ For CV curves, on the other hand, at a given scan rate *v* and film thickness *L*, the measured current density *i* is directly related to the chemical capacitance via
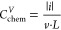
18assuming the scan rate is slow enough for the electrode to remain in equilibrium, that is, spatially homogeneous. For *v* = 0.1 mV/s and *L* = 100 nm, the current density in μA/cm^2^ is equivalent to the chemical capacitance in kF/cm^3^. Values of 4 to 29 kF/cm^3^ result for our films, and these values can then be directly compared to *C*_chem_ values from impedance fits, as shown in Figure 4 and discussed in the following sections.

### Impedance Spectra and Their Analysis

**Figure 5:**
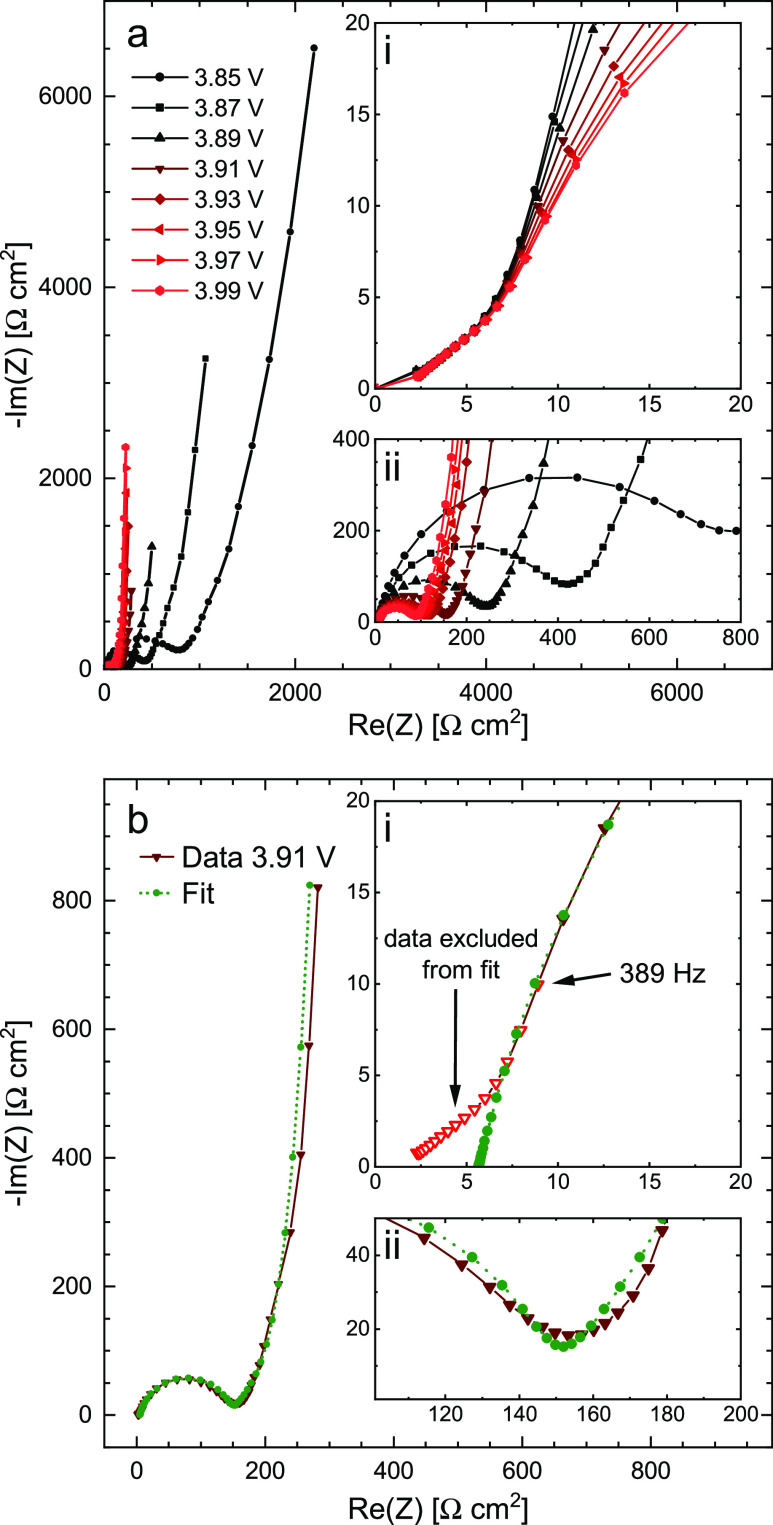
Impedance spectra of the Li_1−δ_CoO_2_ thin film at various electrode potentials. (a) Full spectra with decreasing real impedance in the low-frequency region. The sharp increase and subsequent gradual decrease in chemical capacitance toward higher potentials are indicated by the height of the low-frequency tail. Insets show (i) Warburg-like high-frequency tail of the charge-transfer arc and (ii) inverse variation of the charge-transfer resistance with potential. (b) Exemplary least-squares fit of an impedance spectrum, acquired at an equilibrium electrode potential of 3.91 V vs Li^+^/Li, using a modified Randles’ circuit (Figure 1d). The resulting fit corresponds to the simulated spectrum in Figure 1e. Insets show magnifications of (i) high-frequency region with the onset of the charge-transfer arc and (ii) mid-frequency region with the transition from charge transfer to the solid-state diffusion regime. Measurement points at or above 389 Hz were excluded from the fit and treated as a high-frequency offset.

Figure 5a,b shows the impedance spectra of the LiCoO_2_ thin film in the potential range of 3.85 to 4.00 V versus Li^+^/Li. It should be noted that only every second spectrum is shown for better visibility. The shape of the spectra generally behaves according to the main features of Randles’ circuit (Figure 1e), with the semicircle corresponding to the charge-transfer resistance and a double-layer capacitance, followed by a diffusional tail approaching a purely capacitive behavior toward the lowest frequencies. The most notable difference to the 45–90° behavior expected from the ideal finite-space diffusion element *W*_o_ is that the transition between the semi-infinite and finite-space regimes appears continuous, rather than confined to a specific knee frequency. The charge-transfer resistance and the ionic resistance (as visually estimated by the extrapolated real axis intercept of the diffusional tail) decrease significantly toward higher electrode potentials. A more detailed and quantitative analysis of this part of the spectra is presented below.

At the high-frequency end of the spectrum, a small Warburg-like feature with a real impedance of about 6 Ω is observed that appears independent of the electrode’s SOC. Although we cannot unambiguously assign this feature, it could possibly originate from the substantial surface roughness of the films and some residual porosity or cracks in the thin film. Due to its invariance, small magnitude, and confinement to the high-frequency region, the corresponding frequency points are excluded from the fit, and thus the feature is simply treated as a real axis offset in the following analysis; cf. inset (i) in Figure 5b.

As pointed out in the original literature on finite-space Warburg impedance,^43^ the purely capacitive low-frequency region allows the simultaneous extraction of *R*_ion_ and *C*_chem_ from the impedance spectrum, as the two parameters are completely separated into the real and imaginary parts of the overall impedance. Another direct consequence of this separation is that for a series of impedance spectra over the same frequency range, the height of the capacitive tail in a Nyquist plot directly indicates the capacitance and thus the steepness of the equilibrium charge/discharge curve at the given electrode potential. Indeed, the height of the diffusional tails in Figure 5a,b clearly reflects the shape of the charge curves in Figure 3, with the plateau and hence the highest chemical capacitance around 3.9 V.

In practice, the extraction of *R*_ion_ and *C*_chem_ via CNLS fits of Randles’ circuit can be rather tricky, as the suggested ideal capacitive behavior at low frequencies does not consider nonidealities such as polycrystallinity of the LCO films on Pt, anisotropy of ion conduction in LCO, surface roughness of the films, cracks or residual porosity of the film, and any type of side reactions. Moreover, the frequency range of the measurement is limited by the rapidly increasing measurement times in the low-frequency region. Deviations from ideality can lead to a flattening of the capacitive line below 90° and a smearing out of the transition between 45 and 90°, making it increasingly difficult to achieve reliable fit results. Presumably, due to this reason, previous thin-film studies mostly derived *C*_chem_, or rather d*E*/d*x*, from coulometric titration curves and then used it as a fixed parameter for the impedance fits to extract *R*_ion_ or *D̃*. *C*_chem_, and hence information about the equilibrium charge curve (or differential capacity curve), was directly extracted from the impedance spectra of a Li-ion electrode, for example, in refs (44),^45^, and (71).

In our case, we found that reliable fit results can be obtained by using an anomalous finite-space diffusion element *W*_o_^*^ implemented in the impedance-analyzing software EC-Lab (BioLogic, France) that is similar to the anomalous finite-space diffusion element reported by Bisquert,^47^ yielding the impedance expression
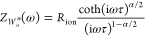
19which allows for a more general power law dependence on time of the mean-squared displacement ⟨*r*^2^⟩ ∝ *t*^β^ rather than the standard linear behavior. More specifically, eq 19 leads to a phase shift ≥45° in the semi-infinite and ≤90° in the finite-space regime, which correctly describes the practical impedance behavior of virtually all thin-film battery electrodes (see Figure 5a).^12,14,15,42^ In our case, the nonideality parameter 0 ≤ α ≤ 1 turned out to be in the range of 0.6–0.7.

As already discussed above, this also means that any conductivity or diffusion coefficient measured in such films must be viewed as an effective rather than a strictly material-intrinsic parameter. Also, normalization of *R*_ct_ to the nominal surface area *A*, that is, without considering the surface roughness or cracks/pores, may somewhat underestimate the true area-specific resistance. Nonetheless, it is fair to assume that the observed general trends, that is, the essential dependences on the electrode potential, remain valid regardless of the film morphology or grain shape and grain size distribution. The impedance expression, as given by eq 19, is substituted for the classical *W*_o_ element into Randles’ circuit. Similarly, the interfacial double-layer capacitance is fitted as a constant-phase element *Q*_dl_ to account for the nonideal capacitive behavior. The modified equivalent circuit used for fitting and its simulated impedance response are shown in Figure 1d,e.

### Fit Results of Elementary Material Parameters

**Figure 6:**
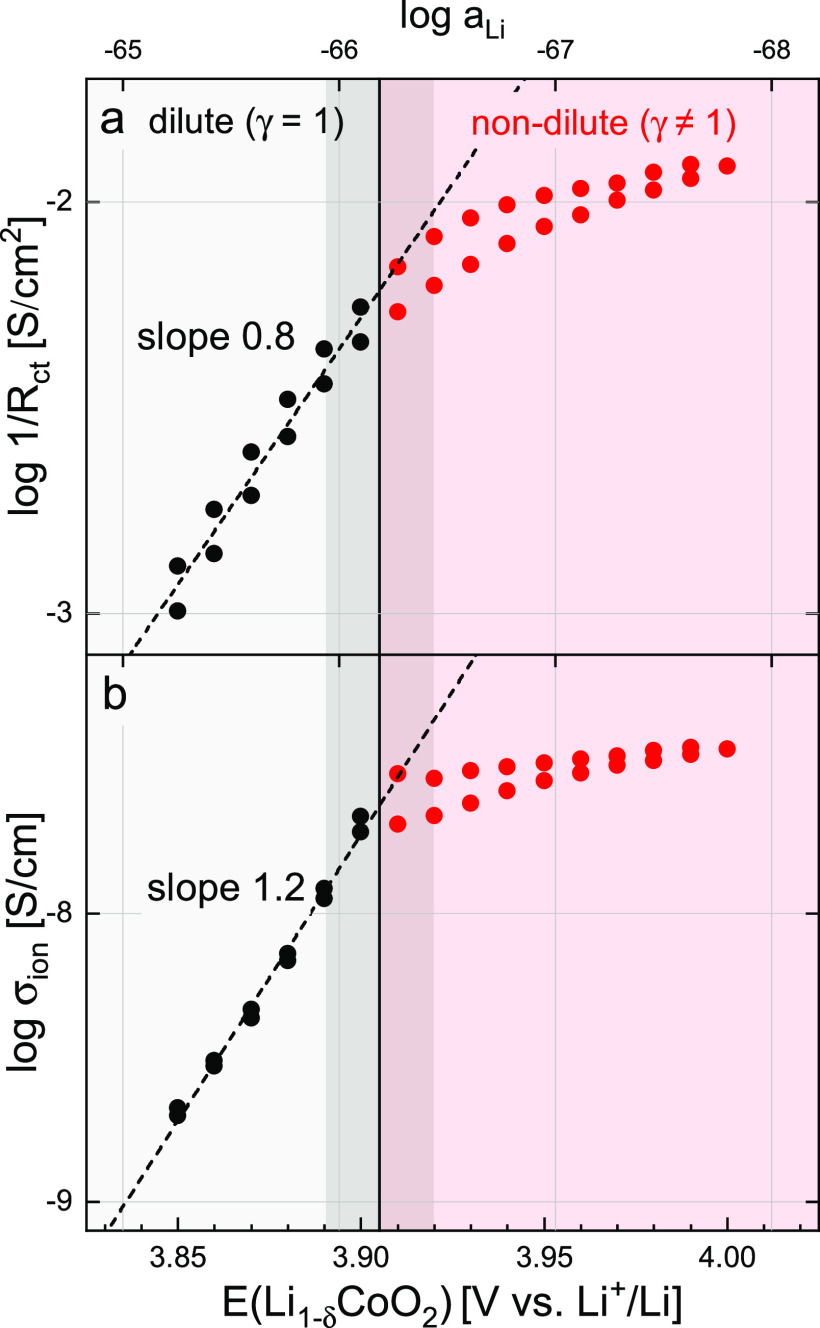
Logarithmic plot of (a) inverse charge-transfer resistance 1/*R*_ct_ and (b) ionic conductivity σ_ion_ vs electrode potential and log *a*_Li_. The dilute (activity coefficient γ = 1) to nondilute transition region 3.89–3.92 V is marked in dark around a central line at 3.905 V. Linear fits for the dilute region are shown as dotted lines.

**Figure 7:**
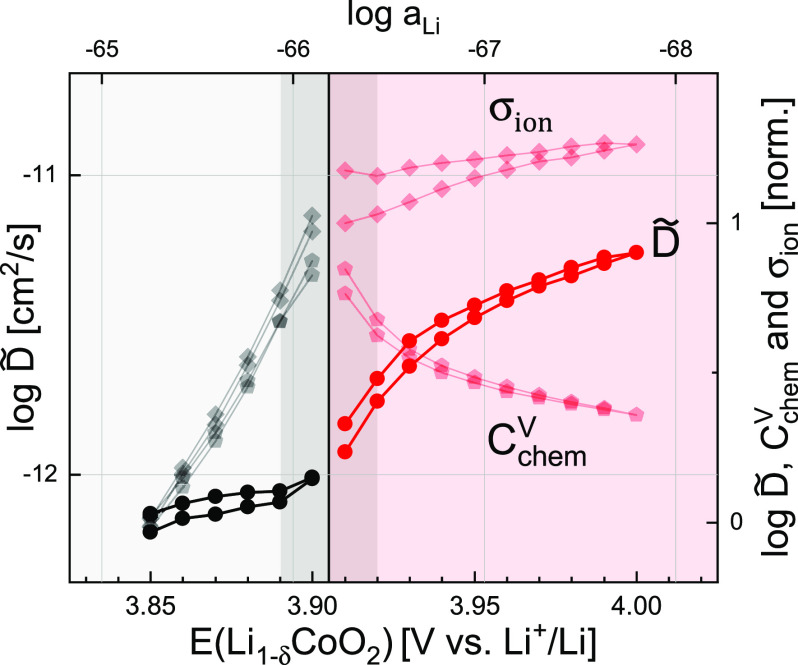
Chemical diffusion coefficient compared to its constituent parameters. The absolute value of *D̃* is given on the left. Values of *D̃*, *C*_chem_^*V*^, and σ_ion_ on the right axis were normalized to their average value at 3.85 V to emphasize their relative trends. The dilute (gray) to nondilute (red) transition region 3.89–3.92 V is marked in dark around a central line at 3.905 V.

As shown by the exemplary least-squares fit in Figure 5b, the equivalent circuit in Figure 1d adequately describes the recorded impedance spectra and therefore allows the extraction of electrochemical properties as a function of electrode potential. The values of 1/*R*_ct_ and σ_ion_ shown in Figure 6a,b, respectively, are directly obtained from the fit and subsequent normalization by sample geometry (*A* and *L*). *C*_chem_ results directly from τ and *R*_ion_ according to eq 17 and is plotted in Figure 4. Finally, *D̃* either results directly from τ in eq 16 or from *C*_chem_ and *R*_ion_ in eq 13, as shown in Figure 7. The parameter values vary by more than 1 order of magnitude and are plotted logarithmically versus the electrode potential and log *a*_Li_ to facilitate their interpretation. The entire dataset shows a minor hysteresis with respect to the scan direction. In the discharge scan starting at 4.0 V, resistances are slightly higher, and chemical capacitances are lower compared to the charge scan. However, these differences are negligible for the overall trends.

The charge-transfer resistance is the highest (about 1000 Ω cm^2^) in the low-potential region and then strongly decreases down to about 100 Ω cm^2^ at 3.99 V, in good agreement with the literature.^12,14,15,42^ At lower potentials, up to ca. 3.9 V, a constant slope of about 0.8 results from the log–log plot of 1/*R*_ct_ versus *a*_Li_. Above a threshold potential of around 3.89–3.92 V, the log–log behavior flattens out. Judging by the values of δ in Figure 3, the corresponding stoichiometry of this transition is approximately Li_0.78–0.92_CoO_2_, although this can only be taken as a rough estimate due to the strong variation of stoichiometry in this potential region. In the following, the low- and high-potential regions separated by this transition will be referred to as dilute and nondilute regimes, respectively.

Effective ionic conductivities strongly increase with the electrode potential, ranging from about 10^–8.7^ S/cm at 3.85 V up to 10^–7.4^ S/cm at 3.99 V. Literature reports of ionic conductivity as a function of stoichiometry are scarce—the only reported values we could find are around 10^–7.5^ to 10^–6.5^ S/cm up to δ = 0.5, although the corresponding dataset does not show the expected decrease of conductivity for low δ.^42^ In the dilute regime, a slope of 1.2 is observed in the log–log plot of σ_ion_ versus *a*_Li_ up to the threshold potential region around 3.89–3.92 V, above which the slope starts to decrease as already seen for 1/*R*_ct_.

The values of *C*_chem_ obtained from the impedance fits are plotted in Figure 4, together with the CV data. Both sets of data are in very good qualitative and quantitative agreement, starting at very small values at low potentials, sharply increasing toward a marked peak of roughly 30 kF/cm^3^ around 3.9 V, and then decreasing to moderate values. The peak absolute values are in excellent agreement with the typical differential capacities found in the literature,^13,14,17,65^ all of the cited values being in the range of 30–40 kF/cm^3^ when normalized by sample geometry. The close match of values from AC and DC data demonstrates the often-overlooked fact, that the properties of a Li electrode’s equilibrium charge curve are fully contained within its potential-dependent impedance response, because both *E* and *C*_chem_ relate back to the fundamental thermodynamic relationship between μ_Li_ and *a*_Li_ in eq 2. Again, a power law dependence between *C*_chem_ and *a*_Li_ can be observed in the dilute regime, this time with a slope of 1.0. A more detailed interpretation of the slope of 1 in terms of defect chemistry is given in the next section.

Finally, the chemical diffusion coefficient, as calculated from eq 16, is shown in Figure 7. As σ_ion_ and *C*_chem_, both of which increase exponentially with *E*, show a similar increase up to 3.9 V, *D̃* remains nearly constant around 10^–12^ cm^2^/s in the dilute regime. Above 3.9 V, *D̃* increases up to almost 10^–11^ cm^2^/s, driven by both an increasing σ_ion_ and a decreasing *C*_chem_. Values below 3.9 V are rarely reported, but the diffusivities at higher potentials are in good agreement with other studies of LCO thin films.^12−15,18,72^

**Figure 8:**
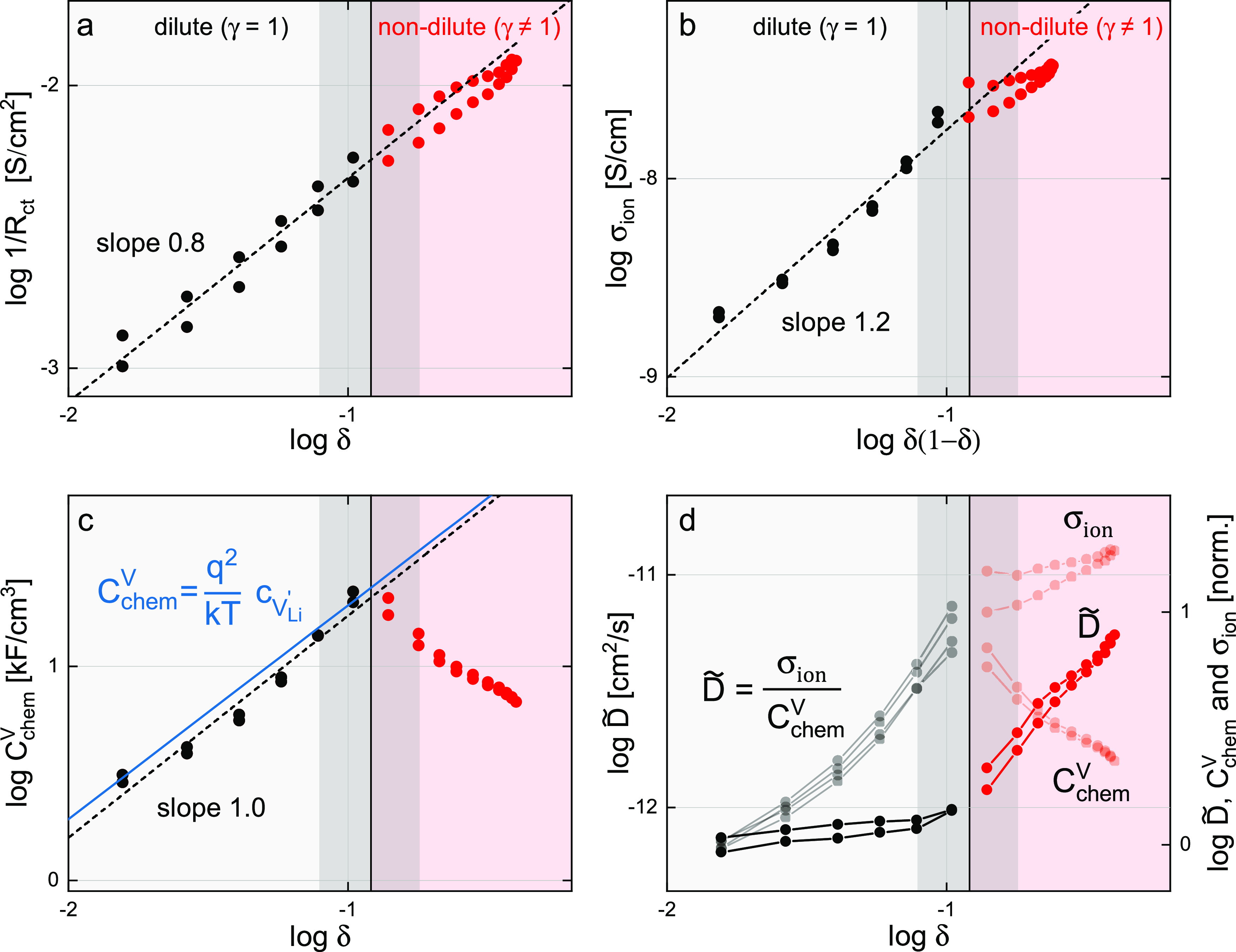
Electrochemical transport parameters of the Li_1−δ_CoO_2_ thin film extracted from impedance spectra shown as log–log plots vs δ (a,c,d) or δ(1 – δ) (b). Values of δ were obtained from Figure 3b by taking the average of all four coulometric titration curves. (a) Inverse charge-transfer resistance, (b) ionic conductivity, and (c) chemical capacitance from impedance fits. The solid blue line indicates the theoretical values predicted by eq 9. (d) Chemical diffusion coefficient compared to its constituent parameters. The absolute value of *D̃* is given on the left. Values of *D̃*, *C*_chem_^*V*^, and σ_ion_ on the right axis were normalized to their average value at 3.85 V to emphasize their relative trends. The dilute to nondilute transition region 3.89–3.92 V is marked in dark around a central line at 3.905 V. Linear fits for the dilute region are shown as a dotted line.

As both the DC data and *C*_chem_ from impedance fits clearly show that the dependence of δ on *E* deviates from a purely exponential behavior in the nondilute regime (i.e., *E* ≠ *E*^0^ + *kT* ln δ^α^, α = const), it is hardly surprising that the corresponding electrochemical properties show a similar deviation. To isolate the concentration dependences from other factors that influence *E*, it is therefore useful to plot all parameters also in dependence of δ. As shown in Figure 8a, the trend of 1/*R*_ct_ straightens, and we get an almost constant slope in the entire range when plotting as a function of nonstoichiometry. In Figure 8b, the ionic conductivity is plotted as a function of δ(1 – δ) rather than δ to account for the limited number of available lattice sites. This also leads to a straightening of the trend, as compared to Figure 6b, with σ_ion_ increasing even toward the highest values of δ(1 – δ), albeit with a smaller slope.

Given the nonidealities of the used impedance model and thus unavoidable uncertainties of the extracted parameters, we consider the slope of σ_ion_ in the dilute regime as close to unity. This suggests that for the dilute regime, ion conduction in LCO can be well described by a vacancy-mediated process with almost constant vacancy mobility. At higher vacancy concentrations (nondilute regime), the slope of σ_ion_ decreases, meaning that the ionic mobility decreases, probably due to some defect interaction. However, still the total conductivity increases with increasing δ, even for very high vacancy concentrations. This is very different from the often-observed conductivity decrease of oxide ion conductors with increasing doping and thus oxygen vacancy concentration (e.g. yttria-stabilized zirconia^73^ or doped ceria^74−76^). Since it is mainly the high concentration of the dopants themselves that reduces the defect mobility in those oxide ion conductors, the reason for the different behavior of our LCO thin films might be the absence of a varying dopant concentration.

The observed log–log dependence of *R*_ct_ on δ (slope 0.8) is in line with a defect chemical picture, where a Li^+^ ion from the electrolyte needs a Li vacancy at the LCO surface for a charge transfer into the electrode. The exact dependence of this charge-transfer reaction on the specific defect concentrations, however, also varies with the concentration dependence of the corresponding Galvani potential step,^77^ and a more detailed discussion is beyond the scope of this paper.

The chemical capacitance in Figure 8c, however, retains its peak in the transition region of the log *C*_chem_ versus log δ plot and strongly decreases in the nondilute regime. In the dilute regime, *C*_chem_ is also proportional to δ and thus to the vacancy concentration. This is in accordance with the expected defect dependence of eq 9, that is, for . As indicated by the blue solid line in Figure 8c, the values of *C*_chem_ predicted by eq 9 are even in excellent quantitative agreement with the experimental data, strongly suggesting that the therein contained assumption  is in fact valid for low vacancy concentrations, as will be discussed in the next section. Furthermore, this striking agreement of theory and experiment demonstrates that the thermodynamic description based on dilute point defects can offer valuable hints at the underlying defect chemistry of Li-ion battery electrode materials and once more highlights the central importance of the chemical capacitance as a powerful, readily accessible material descriptor.

### Defect Chemical Perspective

From all the data presented in Figures 4–8, we conclude that (i) in the dilute regime, log δ is proportional to −log *a*_Li_, with a slope close to unity, and (ii) upon transition to the nondilute regime, the electrochemical properties of LCO collectively change in their dependence on Li activity and electrode potential. This dilute to nondilute transition occurs precisely in the region of the highest chemical capacitance—the plateau of the charge curve—where the stoichiometry strongly varies with the electrode potential and particularly affects properties, which themselves include a dependence on the Li activity (*C*_chem_, *D̃*). Charge transport and transfer (σ_ion_ and *R*_ct_), on the other hand, which primarily depend on the Li vacancy concentration, remain unaffected when plotted versus δ rather than Li activity.

We first consider the dilute regime and discuss the possible defect chemical reasons behind the slopes of *C*_chem_ and σ_ion_ in their respective log–log plots versus *a*_Li_, both being reasonably close to 1. We combine eqs 4–6 and, under dilute assumptions, arrive at
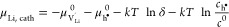
20and thus
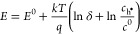
21with
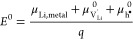
22In accordance with eq 2, we may also write
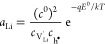
23Assuming negligible defect concentrations due to intrinsic ionic or electronic disorder and the absence of any dopant charge, charge neutrality requires , and eq 21 reduces to
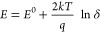
24Similarly, eq 23 leads to
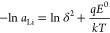
25Equation 25 predicts a slope of 1/2 in a plot of log δ versus −log *a*_Li_, that is, in a Brouwer diagram.

Alternatively, and perhaps more intuitively, the power law dependences of point defect concentrations on activity can also be derived from equilibrium mass action laws.^49^ For the case of Li intercalation into a cathode material, the equilibrium reaction and its corresponding mass action law can be formulated as
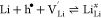
26
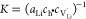
27where *K* is the equilibrium constant of the intercalation reaction. In accordance with eq 25, eq 27 predicts  and therefore a slope of 1/2 in a plot of log δ versus −log *a*_Li_, again assuming  due to charge neutrality.

Instead, we find for σ_ion_ a slope of roughly 1 in the log σ_ion_ versus log *a*_Li_ plot. This somewhat surprising result is very consistent with the potential dependence of *C*_chem_: assuming only defects due to nonstoichiometry (i.e., Li deintercalation, ), we would expect a slope of 1/2 according to eqs 8, 25, and 27. However, the measured slope for *C*_chem_ is also close to 1. Within the thermodynamic description of *C*_chem_ presented above, a slope of 1 could be explained if  or  and assuming the majority carrier concentration as relatively constant on a logarithmic scale. Given the strong variation of *R*_ct_ and σ_ion_, Li vacancies can be assumed to be the minority carriers, and *c*_h^•^_ has to be pinned significantly above  to explain the observed slopes. The excellent quantitative agreement of the experimental values of *C*_chem_ with eq 9 also supports this assumption. If the concentrations of both vacancies and holes were relevant, eq 8 would predict *C*_chem_ values to be lower by a factor 2 compared to those found experimentally.

From a defect chemical perspective, pinning of *c*_h^•^_ might occur if the hole concentration introduced through nonstoichiometry is negligible compared to the levels of either (i) intrinsic electronic disorder or (ii) extrinsic acceptor doping. The evaluation of the former is far from trivial, as Li_1−δ_CoO_2_ is known to undergo a semiconductor–metal transition upon delithiation, accompanied by a significant increase in electronic conductivity and hole mobility as the conduction mechanism transitions from localized polaron hopping toward delocalized metallic conductivity.^60,63,78^ Owing to the close entanglement of mobility and conductivity, it is not entirely clear how *c*_h^•^_ behaves in this stoichiometry range. Furthermore, the defect chemical description of electronic charge carriers relies on the semiconductor-type behavior with thermally activated, localized polarons and therefore fails to describe metallic conductivity.

A more accessible explanation lies in the presence of an extrinsic acceptor dopant in the material. As reported in the literature, the formation energies of antisite defects such as Li_Co_^″^ are relatively low, and the defect chemical composition of nominal LiCoO_2_ thin films is highly sensitive to synthesis conditions.^53,79−81^ It would therefore come as no surprise if the sputter-deposited and post-annealed thin films examined in this work contained a significant amount of Li_Co_^″^ defects that act as acceptor dopants in the material, although further experimental investigation would be required to corroborate this hypothesis.

**Figure 9:**
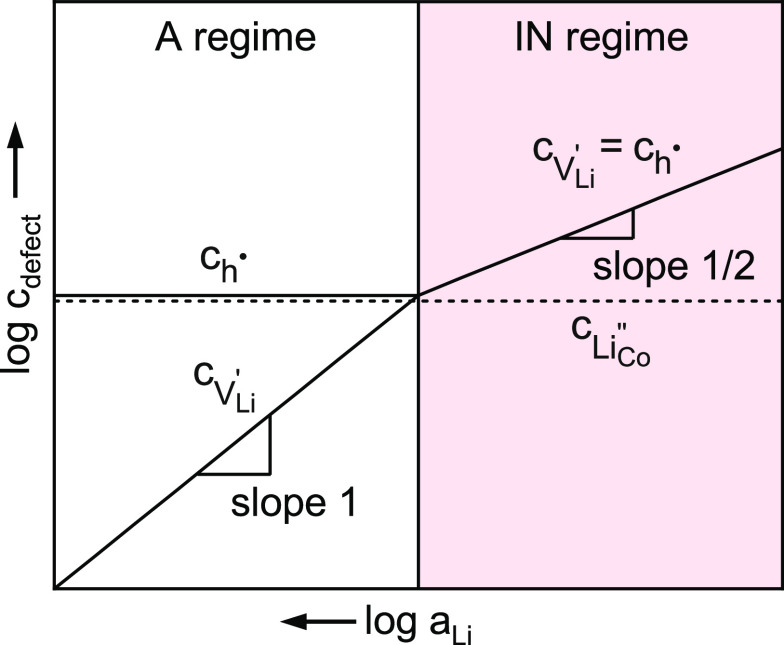
(a) Proposed Brouwer diagram of inadvertently acceptor-doped Li_1−δ_CoO_2_, showing logarithmic defect (vacancy and hole) concentrations as a function of −log *a*_Li_. In the A (acceptor) regime, the electron hole concentration is fixed by the negative acceptor dopant (e.g., Li_Co_^″^) concentration, and . In the IN (ideal nonstoichiometry) regime, the hole and vacancy concentrations due to nonstoichiometry start to dominate, and . In both regimes, defect activity coefficients γ_*i*_ are assumed to be 1.

Based on the assumed presence of an acceptor dopant such as Li_Co_^″^, we can also propose a Brouwer diagram. The dopant requires compensation by a formally positively charged defect species to preserve charge neutrality. For high *a*_Li_ and thus low  (see eq 27), the dopant causes a fixed hole concentration to satisfy the electroneutrality condition . As long as the additional hole concentration introduced through the charging of the electrode is comparatively small, the overall hole concentration will therefore remain near-constant on a logarithmic scale. If *c*_h^•^_ is constant, eq 27 requireseq 27. This situation, labeled as A (acceptor) regime, is shown on the left side of Figure 9 at high lithium activities. Once the initially fixed hole concentration is surpassed by the additional concentrations introduced through nonstoichiometry, the activity dependences of *c*_h^•^_ and  behave according to eqs 24 and 25. The proposed defect model would therefore not only explain the slope of about 1 for σ_ion_ at low δ but would even predict a change of slope toward 1/2 at high δ, where  is valid. As a first estimate, as the flattening of the log–log slopes in Figures 4 and 6 occurs around δ ≈ 0.1, we can thus suggest a Li_Co_^″^ site fraction of about 5%.

Given the low operating temperature and high defect concentrations, as compared, for example, to high-temperature oxygen-ion conductors, one could also expect additional ionic–electronic defect association of vacancies and holes.^49,51^ Although this effect cannot explain the initial slope of 1, it might further enhance the flattening of the slope toward higher δ and is therefore also consistent with our results. Li interstitials, on the other hand, are not expected to play a significant role. These may come into play for a high level of Frenkel disorder, which would lead to *a*_Li_-independent intrinsic vacancy (and interstitial) concentrations, in contrast to the experimental results.

However, under the dilute assumptions implicit in the Brouwer diagram, *C*_chem_ would be predicted to be  in the dilute regime and  beyond—see eq 8. Hence, it should follow the same slopes as σ_ion_. The decrease of *C*_chem_ above 3.9 V is therefore beyond the idealized model presented above and can only be explained by considering the varying activity coefficients of holes and vacancies at high concentrations. As both σ_ion_ and *C*_chem_ necessarily scale with the same concentrations within the dilute model, we can also conclude from eq 12 that the strong increase of *D̃* at high δ can in fact only result from the nonideal, that is, nondilute, behavior. This highlights the fact that, although it may be in line with intuition to see an increase in diffusivity at higher carrier concentrations, the reasons for this experimentally observed behavior are actually far from obvious, given the parallel concentration dependences of σ_ion_ and *C*_chem_. Conversely, a strong variation of diffusivity as a function of SOC can be seen as an indication that the dilute model is no longer sufficient to describe the material’s transport properties.

## Conclusions

A full set of electrochemical parameters describing the mass and charge transport properties of Li_1−δ_CoO_2_ thin films could be simultaneously extracted from impedance spectra measured in dependence of the SOC. The relevant elementary material parameters are the ionic conductivity σ_ion_, charge-transfer resistance *R*_ct_, and chemical capacitance *C*_chem_. It is shown that the chemical capacitance can be deduced alternatively from DC data (coulometric titration curve or CV), and both approaches lead to very consistent results. For a dilute regime (δ < 0.1), the chemical capacitance is even in excellent quantitative agreement with defect chemical predictions. This demonstrates the central importance of the chemical capacitance as a powerful, readily accessible material descriptor. The Li chemical diffusion coefficient, on the other hand, is a composite property, and its dependence on the SOC can only be understood from the underlying ionic conductivity and chemical capacitance. By evaluating the dependence of all elementary material parameters on Li activity and nonstoichiometry, it was shown that at low potentials, that is, low vacancy concentrations, the transport properties of the investigated Li_1−δ_CoO_2_ thin film are consistently described by a dilute defect chemical model. However, the analysis of slopes in the log–log plots of σ_ion_ and *C*_chem_ versus Li activity, as well as the absolute values of *C*_chem_, strongly suggests the presence of an acceptor dopant. Li_Co_^″^ antisite defects, inadvertently introduced during sputter deposition, are proposed as the plausible acceptor species. As a result, both *C*_chem_ and σ_ion_ increase exponentially with the electrode potential (up to δ ≈ 0.1), and *D̃* remains nearly constant. At high potentials, that is, high vacancy concentrations, the dilute model fails as activity coefficients start to become relevant and *C*_chem_ begins to decrease. The measured increase of *D̃* for δ > 0.1 can therefore serve as an indicator of the material moving beyond the ideal dilute behavior. The ionic conductivity, however, increases almost linearly in the corresponding log–log plot versus δ(1 – δ) for the entire stoichiometric range, which indicates little concentration dependence of the vacancy mobility.
